# Evaluation of a digitized physician-patient-communication course evaluated by preclinical medical students: a replacement for classroom education?

**DOI:** 10.3205/zma001378

**Published:** 2020-12-03

**Authors:** Sabine Fischbeck, Jochen Hardt, Camila Malkewitz, Katja Petrowski

**Affiliations:** 1 Universitätsmedizin Mainz, Klinik und Poliklinik für Psychosomatische Medizin und Psychotherapie, Medizinische Psychologie und Medizinische Soziologie, Mainz, Germany

**Keywords:** online-education, physician-patient communication, medical students, web-based learning, E-learning

## Abstract

**Objectives: **The limitations in teaching resulting from the Covid-19 epidemic were the rational for transferring the course in Medical Psychology and Medical Sociology (doctor-patient communication) into an asynchronous e-learning course. For this purpose, ten exercises were developed to be downloaded by the students and the solutions returned to the course lecturer on a weekly basis. In addition, two students individually recorded via video one of eight doctor-patient exercise conversations, which were then evaluated by four other students and the respective lecturer.

**Methods: **For evaluation, the students filled out an exercise and an effect-related questionnaire with 21 items.

**Results: **The questionnaire was completed by n=203 (98%) students (59% female, 41% male). The video-based situation analyses (91%) helped most of them to become rather closely or very well acquainted with medical conversation practice. 76% rated the exercise “Enlightenment Conversation/SPIKES Protocol” as fairly helpful or very helpful in respect to the practicing concepts of medical conversation. When asked about the effects, most of them found the idea of patient orientation in medicine to be quite helpful or very helpful (83%). About a quarter of them (24%) stated that the online course could not, or only slightly, replace face-to-face teaching. This assessment was less pronounced among female students than among male students (Wilcoxon test p<.01).

**Conclusion: **Our online course concept of physician-patient conversation found good overall response among pre-clinical medical students. However, the participants expressed different opinions about the extent to which the concept can replace face-to-face teaching.

## Zusammenfassung

**Zielsetzung: **Die sich aus der Covid-19 Epidemie ergebenden Beschränkungen in der Lehre waren Grund, den Kursus der Medizinischen Psychologie und Medizinischen Soziologie (Arzt-Patient-Kommunikation) in einen asynchronen E-Learning Kursus zu überführen. Für diesen Zweck wurden zehn Übungsaufgaben entwickelt, welche die Studierenden wöchentlich herunterladen und die Lösungen an den Kursdozenten zurückschickten. Zusätzlich zeichneten je zwei Studierende eines von acht Arzt-Patient-Übungsgesprächen auf, das von vier anderen Studierenden sowie dem jeweiligen Dozierenden bewertet wurde.

**Methodik: **Zur Evaluation füllten die Studierenden einen übungs- und effektbezogenen Fragebogen mit 21 Items aus.

**Ergebnisse: **Den Fragebogen bearbeiteten n = 203 (98%) der Studierenden (59% weibliche, 41% männlich). Für die meisten hatten die videobasierten Situationsanalysen (91%) ziemlich/sehr gut die Nähe zur Praxis der Ärztlichen Gesprächsführung hergestellt. Die Übung „Aufklärungsgespräch/SPIKES-Protokoll“ beurteilten 76% als ziemlich oder sehr hilfreich, um Konzepte der Ärztlichen Gesprächsführung einzuüben. Gefragt zu den Effekten sahen die Meisten den Gedanken der Patientenorientiertheit in der Medizin ziemlich oder sehr befördert (83%). Etwa ein Viertel (24%) gaben an, der Online-Kursus könne nicht oder nur wenig die Präsenzlehre ersetzen. Bei weiblichen Studierenden war diese Einschätzung weniger ausgeprägt als bei dem männlichen (Wilcoxon-Test p<.01).

**Schlussfolgerung: **Unser Online-Kurskonzept der Arzt-Patient-Gesprächsführung hat bei vorklinischen Studierenden der Medizin insgesamt eine gute Resonanz gefunden. Allerdings sind die Teilnehmer-/innen gespalten, inwieweit das Konzept die Präsenzlehre ersetzen kann.

## 1. Introduction and problem definition

The Covid-19 pandemic has made it necessary to convert face-to-face teaching of university medical courses in Mainz into digital teaching also in the subject Medical psychology and Medical sociology. This causes a particular challenge to the subject course, which is taught in the second semester of pre-clinical studies. Formerly, medical conversation had been taught and tested face-to-face (by means of Situational Judgement Test or Objective Structured (Pre-)Clinical Examination) in small groups of 15 participants (10 appointments, 95 minutes each; see table 1 (Tab. 1) for topics) [1]. Since the switch to digital teaching, however, on site role-plays or engaging simulation patients were no longer possible. Planning concepts from other faculties were hardly available and, so far, only a few, purely web-based course examples for training doctor-patient conversation may be obtained. From the point of view of erstwhile course participants, the former approach led to a subjective gain in competence [2] and to demonstrably improved communication skills [3], [4] in contrast to the usually given disadvantages of depending web-based programs, such as social isolation, de-individualized instruction as well as technical problems [5]. Our goal, therefore, has become to use the now web-based course to convey communicative action and process knowledge employing case-based simulative environments supported by learning management systems [6] and to determine the resonance and subjective learning gain.

## 2. Online course concept

From the former classroom concept of the course, we had access to scripts, textbook chapters, presentation slides in Power Point^®^ format as well as role scripts and corresponding checklists. Since there was little time for the transfer to an online concept, we kept to the ten course units regarding the topic structure and the distribution of the literature. The script as well as a topic- and literature plan could be downloaded by the students even before the course started. For each course unit, the existing slides on communication concepts were uploaded week after week to the ILIAS platform with and without sound track. In addition, we included practice-oriented exercises (4-7 pages) which the students had to work out and return to the lecturers within four days. Most of the exercises were designed as situational judgement test, e.g., formulating answers to patient statements, suggesting improvements of communication on the basis of a doctor-patient photograph or video. Optimal solutions were made available after the end of the processing time. In order to rehearse the medical conversation, the students were given the task of making an appointment with another student via SKYPE, for example, and recording and sending back a doctor-patient simulation conversation according to role specifications (5-10 min, 230 recordings in total). Four (additional) students and the lecturers each gave (peer) feedback using task-related checklists. The topics of the course are shown in table 1 (Tab. 1). At the beginning of the course there was a virtual meeting between the students and the respective lecturer, who could also be contacted later in case of queries.

## 3. Methodology

As a theoretical framework, the extended concept of “Responsive Evaluation” by Heim and Thommen [7] seemed most advantageous. As an evaluation qua resonance, it leads to the students’ judgement of how to assess the specific components and effects of the online concept with regard to achieving the communicated learning goals. A corresponding ad hoc questionnaire refers to the practical relevance of the exercises (5 items), the assistance in practicing the concepts of medical conversation (10 items), and the effects of the online course on achieving the learning goals (6 items). The students were able to indicate on a 5-level Likert scale (1=not, 2=little, 3=medium, 4=rather, 5=very) how good they thought the above-mentioned aspects were in each case. The questionnaire was attached to the last exercise. Descriptive statistics were used for the evaluation; gender differences in the effect items were also evaluated, using the Wilcoxon rank test, whereby it was assumed [4] that the online concept would be rated more highly r by male students than by female students.

Of the n=208 students in the semester, n=203 (98%) filled out the evaluation questionnaire. Of the n=148 who named their semester, 63% (age M=24.2 years, n=116/59% female, n=116/56%) were single/living by themselves in the second semester. 59% (n=116) had completed their medical training, most of them as nurses (n=50, 43%).

## 4. Results

Asked about how well the task forms in the ten exercises produced the proximity to the practice of medical conversation or competence, the video-based situation analyses rated the highest, as can also be seen from table 1 (Tab. 1): here, 91% reported that they had been rather good/very good.

The exercise “Enlightenment Conversation/SPIKES Protocol” for practicing medical conversation was found to have been fairly/very helpful by 76 %, and somewhat less so for “Stress and Stress Management” (60%). 

In terms of gaining competence and achieving the goals of the online course, most felt that the exercises had rather/strongly promoted the idea of patient-orientation in medicine (83%) and also brought about progress in medical psychological knowledge (70%). About a quarter (24%) felt that the online course could not or could only slightly replace classroom teaching. This assessment was significantly less pronounced among female students than among male students (w: M=3.43, SD=1.19, m: M=3.01, SD=1.17; Wilcoxon test p<.01). The latter also found that the course had promoted the idea of patient-orientation in medicine more strongly (w: M=4.38, SD=.74, m: M=4.06, SD=.74; Wilcoxon test p<.001).

## 5. Conclusion

From the point of view of the students, the transfer of a preclinical compulsory face-to-face course of doctor-patient communication into an online format presents itself altogether as highly satisfactory regarding practice orientation, quality of exercises, and authority profit. However, some of the students and – unexpectedly (compared to Peksen) [8] – especially the male students stronger do not find the online format as an adequate substitute for classroom teaching. Our evaluation cannot answer which learning needs could not be met by the course; nevertheless, we hope to discover this in Winter Semester 2020/21. In how far our web-based concept is demonstrably suitable for promoting the communicative competencies of students to the same extent as classroom teaching should be tested in an experimental approach in the future. At least supplementing classroom teaching in a blended learning approach with web-based components seems to be a favorable strategy for the acquisition of action knowledge on the level of know-how. Whether online recordings of doctor-patient simulation conversations and the corresponding checklists’ feedback will lead to similar learning benefit as working in small groups is another research desideratum. Overall, the positive feedback from students encourages us to stay with our online concept as long as necessary.

## Competing interests

The authors declare that they have no competing interests. 

## Figures and Tables

**Table 1:**
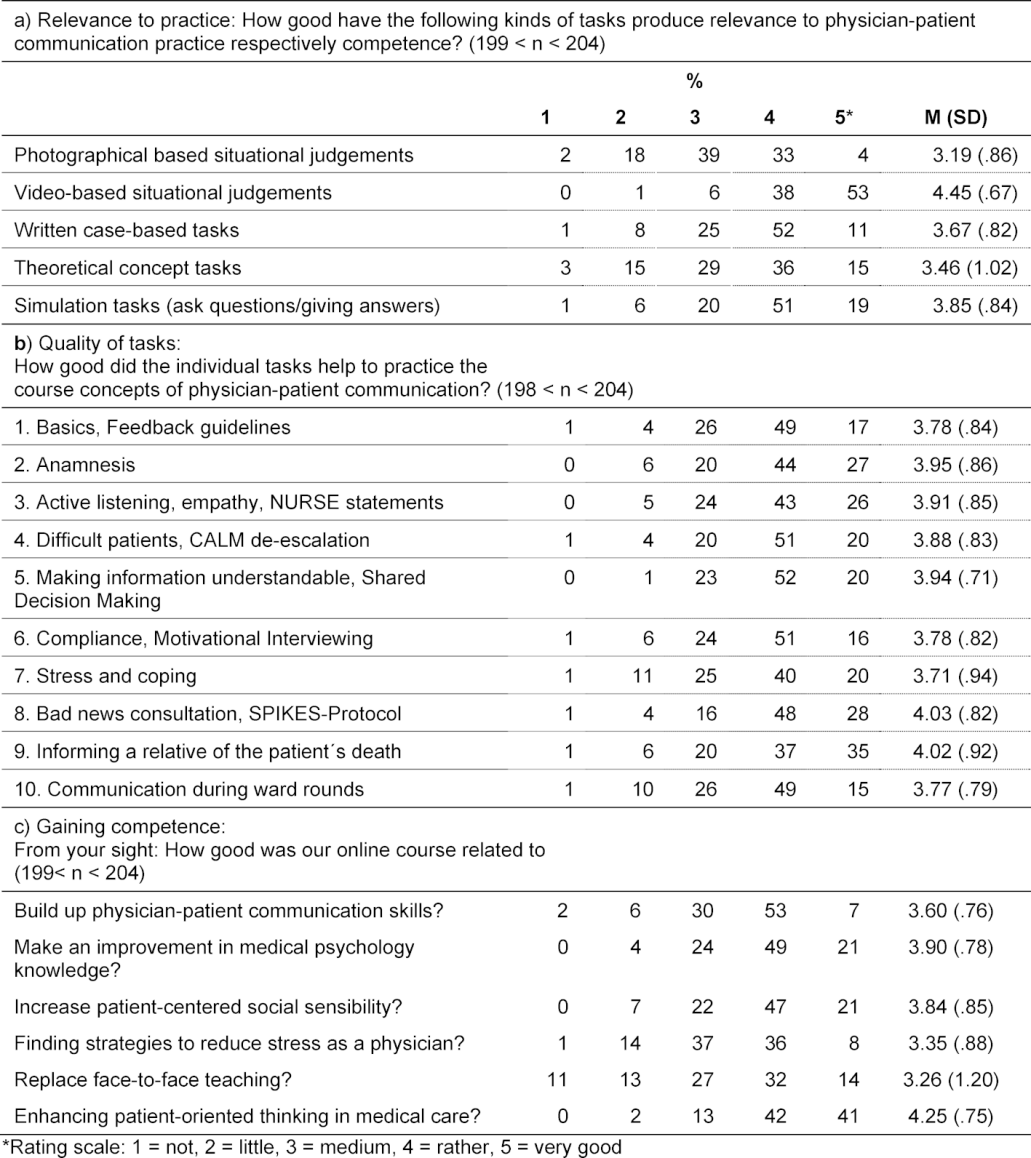
Evaluation of the Online-Course “Physician-patient communication”
